# Incidence, symptom clusters and determinants of post-acute COVID symptoms: a population-based surveillance in community-dwelling users of the COVID RADAR app

**DOI:** 10.1136/bmjopen-2024-087235

**Published:** 2024-09-10

**Authors:** Willian J van Dijk, Miriam L Haaksma, Dennis O Mook-Kanamori, Leo G Visser, Mattijs E Numans, A van Hylckama Vlieg, Frits R Rosendaal, Jessica C Kiefte-de Jong

**Affiliations:** 1 Department of Public Health and Primary Care, Leiden University Medical Center, Leiden, The Netherlands; 2 Department of Clinical Epidemiology, Leiden University Medical Center, Leiden, The Netherlands; 3 Department of Infectious Diseases, Leiden University Medical Center, Leiden, The Netherlands

**Keywords:** COVID-19, vaccination, post-acute COVID-19 syndrome, community participation

## Abstract

**Abstract:**

**Objectives:**

This study aims to describe the incidence, symptom clusters and determinants of post-acute COVID symptoms using data from the COVID RADAR app in the Netherlands.

**Design:**

Prospective cohort.

**Setting:**

General population in the Netherlands from April 2020 to February 2022.

**Participants:**

A total of 1478 COVID RADAR app users, with data spanning 40 days before to 100 days after positive SARS-CoV-2 test.

**Outcome measures:**

Incidence and duration of 10 new symptoms that developed during acute infection, defined as 10 days prior and 10 days after positive test. Clustering of these post-acute COVID symptoms and associations between factors known in the acute phase and 100-day symptom persistence.

**Results:**

The most frequent post-acute symptoms were cough, loss of smell or taste and fatigue. At 100 days postinfection, 86 (8%) participants still experienced symptoms. Three post-acute COVID symptom clusters were identified: non-respiratory (headache and fatigue; 49% of participants with post-acute COVID symptoms); olfactory (15%) and respiratory (8%). Vaccination was associated with a lower risk of post-acute COVID symptoms 100 days after infection*,* although CIs were wide (OR: 0.5; 95% CI: 0.2 to 1.5), but not with non-respiratory symptoms (OR: 1.0; 95% CI: 0.3 to 4.4). Severe acute disease increased the risk of post-acute COVID symptoms (OR: 1.4; 95% CI: 1.2 to 1.5; per additional acute symptom).

**Conclusions:**

In this cohort of infected community-dwelling app users, 5%–10% experienced post-acute COVID symptoms. The symptoms cluster in several distinct entities, which differ in incidence, patient characteristics and vaccination effects. This suggests multiple mechanisms underlying the development of post-acute COVID symptoms.

**Strengths and limitations of this study**

- This study uses data from community-dwelling participants.
- This study was able to measure newly developed symptoms on the individual level, by taking prior symptoms into account.
- Detailed data collection that allowed adjustment for several possible confounders.
- Participation was based on self-selection, which could result in oversampling of users experiencing symptoms.

## Introduction

 In the past years, millions of people have been infected with SARS-CoV-2.^1^ An infection with SARS-CoV-2 can be followed by long-lasting symptoms, with substantial impact on life. These long-lasting symptoms are referred to as ‘post-COVID’, ‘Post-acute sequelae of COVID-19’ or ‘long Covid’, with fatigue as most frequently reported symptom.^2^

Several hypotheses about the pathogenesis of post-COVID include persistent presence or reactivation of viruses, tissue damage, autoimmunity or changes in the microbiome.^3^ But also endothelial activation, coagulation activation and the formation of neutrophil extracellular traps are proposed mechanisms of post-COVID.^4^,^7^

Research on post-COVID is challenging. Studies that have been performed on the subject varied in selection (many based on clinical cohorts), and length of follow-up. This heterogeneity, and that of definitions of post-COVID regarding type, duration, number and severity of symptoms, has led to a wide range of prevalence estimates, from 5% to 50%.^8 9^ In addition, several subtypes of post-COVID have been proposed, increasing the complexity of studying these symptoms.^10^

The WHO defines post-COVID as symptoms not otherwise explained, persisting longer than 2 months following COVID-19 diagnosis in the past 3 months.^11^ WHO emphasised that the definition is temporary as it is based on analyses of small studies with short follow-up in mostly hospitalised patients. Hence they advise to obtain new evidence from prospective studies with sufficient follow-up time, in less selected patient groups such as in primary care and community-dwelling people. An ideal study would be a large prospective longitudinal cohort of community-dwelling people with a sufficient number of repeated measurements for each patient before and after a SARS-CoV-2 test result.

In response to this, we used data from the COVID RADAR smartphone app in the Netherlands, active from April 2020 until February 2022, with which users anonymously answered a short daily questionnaire about their symptoms, SARS-CoV-2 test results and vaccination status.^12^ Using these data, we were able to distinguish newly developed symptoms during acute SARS-CoV-2 infection from pre-existing symptoms.

In this study, our main objective was to identify symptoms persisting beyond the acute phase (post-acute COVID symptoms). Second, we aimed to identify clusters of post-acute COVID symptoms, that is, possible subtypes of Post-COVID, and we investigated which factors in the acute phase (such as severity of disease and vaccination status) were associated with (clusters of) symptoms persisting at least 100 days after a positive test.

## Methods

### COVID RADAR app

The COVID RADAR app was a free app through which users were asked to anonymously report on 10 different COVID-19-related symptoms by filling in a short daily questionnaire, with questions such as ‘Did you cough?’ or ‘Did you have a fever?’^12^ In addition, users gave information about SARS-CoV-2 test results and vaccination status. See online supplemental table 1 for details about the questions and other collected variables. Participation in the app was voluntary; allowing participants to start, pause or stop using the app at their discretion. Different national (social) media campaigns encouraging usage of the app resulted in 284 000 individual users who filled out the questionnaire more than 8.5 million times between April 2020 and February 2022.

On first use of the app, users are asked to provide informed consent to share the information with the research institution. See the online supplemental material: Methods (details) and prior publication for more details.^12^

### Patient and public involvement

Several focus group interviews and qualitative thematic analysis on end-user emails were conducted.^13^ Based on the experiences and feedback of these users, we made several adjustment to the app. The app was dynamic, which allowed for updating questions in response to changes, for instance, changes in mitigation measures, but also improvements in user experience.

### Definitions

We defined the acute phase of COVID-19 as the period between 10 days prior and 10 days after a report of a positive SARS-CoV-2 test. An acute symptom was defined as a symptom reported at least once during the acute phase.

The prior phase of COVID-19 was defined as the period between 40 and 11 days prior to a positive test result (see online supplemental figure 1). A ‘prior symptom’ was defined as a symptom reported at >50% of a participant’s available observations during the prior phase. A symptom was considered ‘new’ if it developed during the acute phase, but was not a ‘prior symptom’.

For each symptom the day of recovery was defined as the day when this symptom was not reported by the participant in 14 consecutive days. The duration of symptoms was calculated for each symptom as the number of days between the onset of the symptom in the acute phase and the first day of recovery from the symptom. If this duration lasted longer than the ‘acute phase’, these symptoms were considered a ‘post-acute COVID symptom’.

### Inclusion criteria

We included participants who reported their first positive test and had at least three prior app entries. They needed to answer the questionnaire for at least 100 days after their positive test or until they fully recovered from all new symptoms within those 100 days. Participants were considered lost to follow-up if they did not report for 14 days. We excluded those who were lost to follow-up before 100 days after positive test and were not recovered at their last report in the app. Since 26 October 2020 (7 months after the launch of the app), the symptoms ‘fatigue’ and ‘headache’ were added to the questionnaire. Given that these two symptoms were frequently reported in prior research as post-acute COVID symptoms,^11^ we included only participants with reports of positive SARS-CoV-2 tests after 5 December 2020 (40 days after 26 October) in the present study, so all included participants could report all symptoms in the ‘prior phase’ and ‘acute phase’.

### Statistical analyses

To describe the incidence and duration of symptoms developed during the acute SARS-CoV-2 infection (new symptoms), we used histograms and median durations.

A correlation matrix was used to analyse which new post-acute COVID symptoms were associated with each other (eg, symptom clusters). Correlations were based on the durations until recovery of each new symptom and clustered using agglomerative hierarchical clustering, with a complete linkage method. For this analysis, data of participants were used when at least two symptoms were present for at least 15 days, to confirm they had symptoms lasting longer than the acute phase.

To analyse which factors in the acute phase of SARS-CoV-2 were associated with post-acute COVID symptoms, we focused on post-acute COVID symptoms that lasted until 100 days after SARS-CoV-2 test result. We included participants with a positive test between 6 December 2020 and 20 November 2021 (100 days before the end of data collection). We performed multivariate logistic regression analyses with the outcome ‘persistence of any new post-acute COVID symptom at 100 days after test result’ and with the outcome ‘persistence of only post-acute COVID symptoms from one of the symptom cluster at 100 days after test result’ (from the previous research question).

For the association between vaccination and new post-acute COVID symptoms at 100 days after test result, we estimated odds ratios (adjusted for sex, age, livability index and period of infection). The primary aim of vaccination is to prevent severe COVID-19. It is likely that the mechanism by which vaccination affects persistence of post-acute COVID symptoms, might be through the effect of vaccination on severity of acute disease (mediation, see online supplemental figure 2). To investigate this, we first used linear regression to assess the association between vaccination and number of newly developed acute symptoms (severity) and second we used logistic regression to assess the association between number of newly developed acute symptoms and persistence of new post-acute COVID symptoms. Subsequently, the possibility of a mediating effect of severity of disease in the association between vaccination and persistence of new post-acute COVID symptoms was assessed by adding the number of newly developed acute symptoms to the model and compare the association between vaccination and persistence of new post-acute COVID symptoms with and without this adjustment. A difference between these associations indicates a possible mediating effect. In analyses with ‘number of newly developed acute symptoms’ we used only participants with at least one newly developed symptom in the acute phase.

The definitions of a ‘prior symptom’ (symptom reported at >50% of a participant’s available observations during the prior phase) and recovery (no symptom in the 14 consecutive days) were based on clinical judgement and not on prior literature. We performed several sensitivity analyses with variations of abovementioned definitions of prior symptoms and recovery or with different lengths of follow-up. See online supplemental material: Sensitivity analyses for details about these analyses. All statistical analyses were carried out using STATA V.16.1, with the exception of the clustering analysis which was performed using Python (using the Scipy package). Data are accessible on https://doi.org/10.17026/dans-zcd-m9dh.^14^

## Results

**Figure 1:**
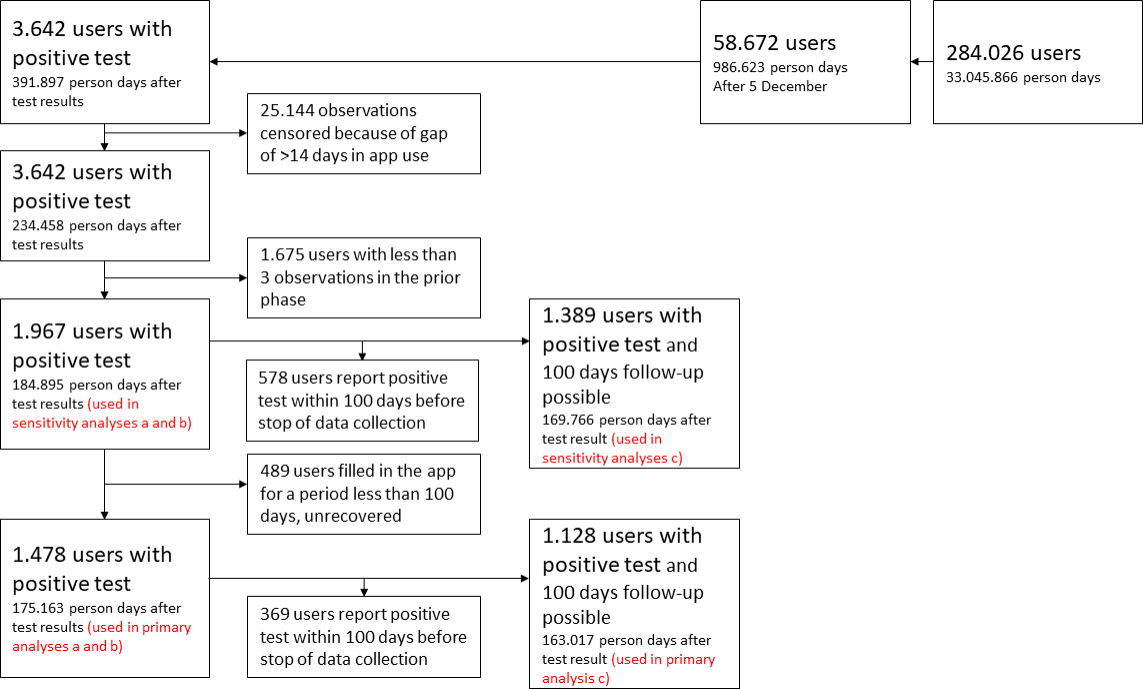
Flow chart of inclusion.

During the research period 58 672 participants used the app (with in total 986 623 person days of data). From these, 3642 participants reported a first positive test result of whom 1478 participants met the inclusion criteria (see figure 1). A total of 1675 participants filled in the app fewer than three times in the prior phase and an additional 489 participants filled in the app for a period less than 100 days and stopped using the app before their recovery. Of those included 865 (59%) were female, 859 (58%) were 60 years or older and 614 (42%) were fully vaccinated before infection. Excluded participants were younger (709 (33%) were 60 years or older) and more frequently vaccinated (1081 (50%); see online supplemental table 2). The pattern of app use is shown in online supplemental figure 3.

### New symptoms after acute SARS-CoV-2 infection and their duration

**Figure 2:**
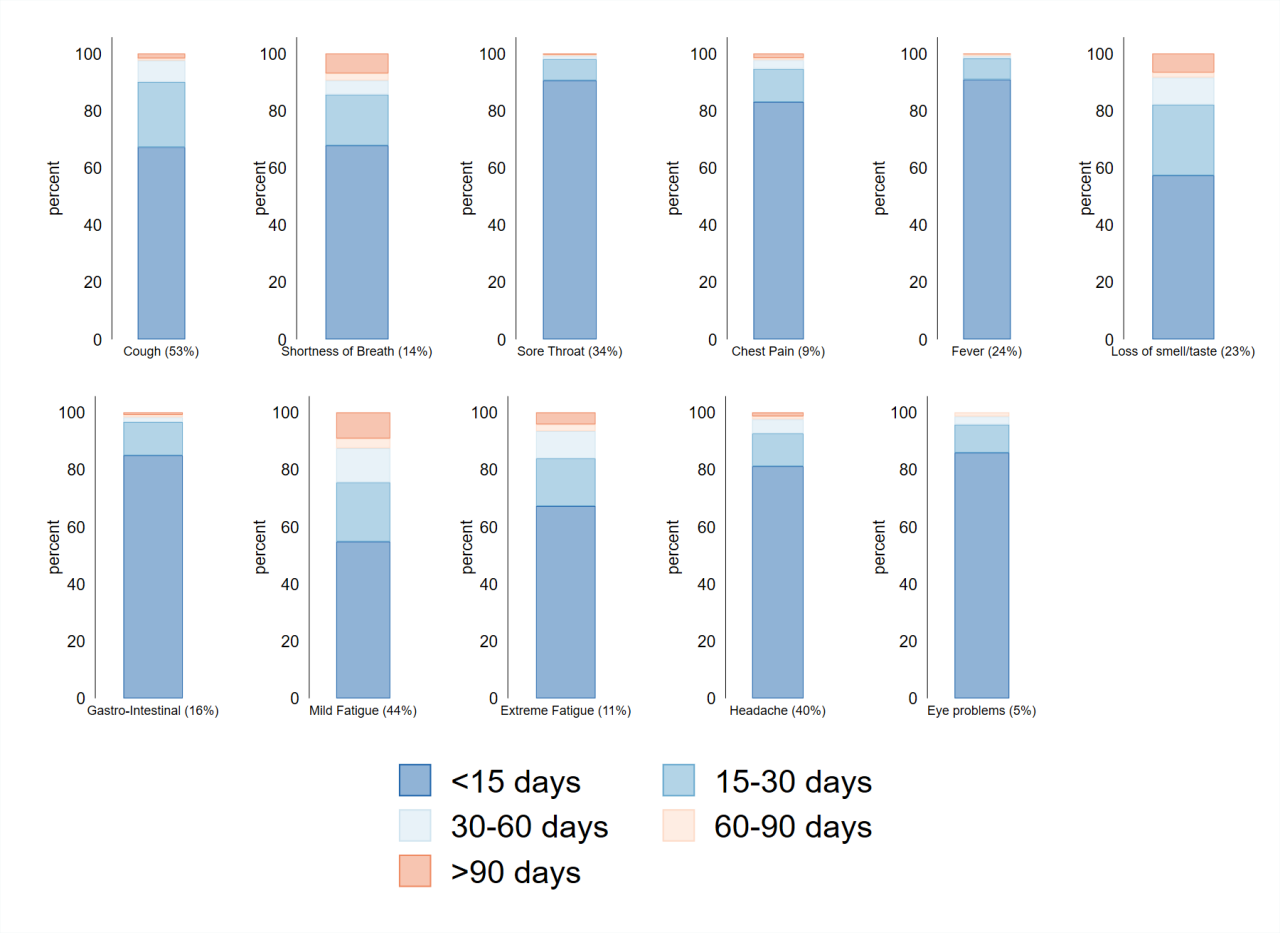
Duration of new symptoms. Example: 783 (53%) of 1478 participants experience coughing during the acute phase (but not during the prior phase), of whom 65% (n=509) recovered within 15 days.

The majority of participants had at least one newly developed symptom (1097, 74%). The most frequently reported symptom was cough (776, 53%). Symptoms that most frequently lasted longer than 90 days were shortness of breath, loss of smell or taste and fatigue (see figure 2 and online supplemental table 3).

### Clustering of post-acute COVID symptoms

**Figure 3:**
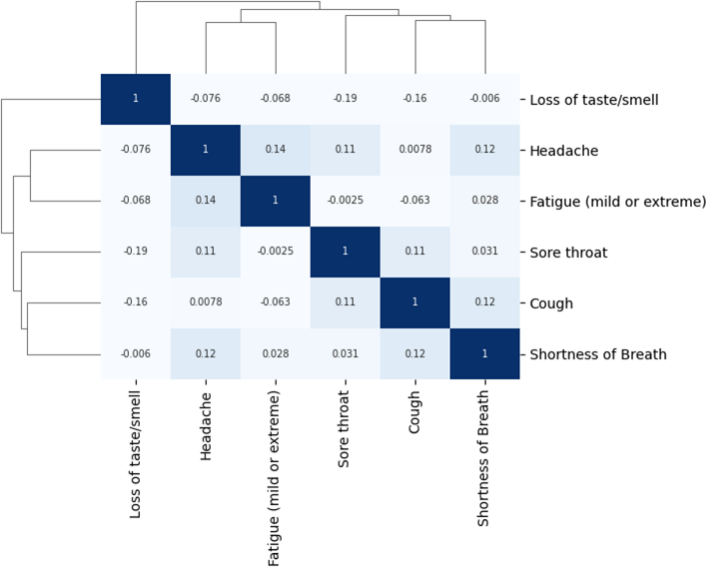
Correlation heatmap. Spearman correlations between duration of post-acute COVID symptoms in participants with at least two symptoms lasting for over 15 days. Using agglomerative hierarchical clustering with a complete linkage method three post-acute COVID symptom clusters were identified: ‘olfactory symptoms’, ‘non-respiratory symptoms’ (fatigue and headache) and ‘respiratory symptoms’.

For 447 participants at least two symptoms lasted for over 15 days. Correlations between duration of post-acute COVID symptoms were low (see figure 3). The symptom ‘Loss of taste or smell’ (ie, olfactory symptoms) showed low correlations with all other post-acute COVID symptoms; and was subsequently clustered as a separate entity. The symptoms ‘fatigue’ and ‘headache’ (ie, non-respiratory symptoms) had highest correlations, and were also clustered. ‘Headache’ was also correlated with some post-acute COVID respiratory symptoms. The respiratory symptoms (‘cough’, ‘sore throat’ and ‘shortness of breath’) were correlated and formed the third cluster of post-acute COVID symptoms.

The sensitivity analysis including the participants without recovery before loss to follow-up and assuming recovery at day of loss of follow-up (n=519), showed similar results (online supplemental figure 4).

### Determinants associated with post-acute COVID symptoms at 100 days

Among the 1478 participants, 1128 reported a positive test before 20 November 2021 (100 days before end of data collection). Of these, 86 (7.8%) had new post-acute COVID symptoms persisting 100 days after a positive test. The proportion of patients with post-acute COVID symptoms was higher in the group infected during the period the alpha variant was most prevalent than during the delta variant period (proportion post-acute COVID symptoms at day 100 after infection with alpha variant: 8.7% vs 4.1% after delta variant). Patients with post-acute COVID symptoms persisting beyond 100 days were mostly female (61 (71%) with post-acute COVID symptoms vs 603 (58%) without post-acute COVID symptoms), of young age (47 (55%) vs 451 (43%) aged under 60 years), more often unvaccinated (76 (88%) vs 779 (75%)) and living in areas with a lower mean livability index (mean Z-score of 0.3 vs 0.5; see table 1).

**Table 1:** Characteristics of infected patients at the acute phase by new post-acute COVID symptoms at 100 days

Post-acute COVID symptoms at 100 days	No	Yes
Number	1042 (100%)	86 (100%)
Sex		
Female	603 (58%)	61 (71%)
Age		
≤18	45 (4.3%)	2 (2.3%)
19–39	55 (5.3%)	8 (9.3%)
40–59	351 (34%)	37 (43%)
>60	591 (57%)	39 (45%)
Vaccinated	263 (25%)	10 (12%)
Time of infection		
Period 1 (November 20–June 21)	787 (91%)	75 (8.7%)
Period 2 (July 21–November 21)	255 (96%)	11 (4.1%)
Vaccination and time of infection		
Period 1 (November 20–June 21) vaccinated	34 (94%)	2 (5.6%)
Period 1 (November 20–June 21) unvaccinated	753 (91%)	73 (8.8%)
Period 2 (July 21–November 21) vaccinated	229 (97%)	8 (3.4%)
Period 2 (July 21–November 21) unvaccinated	26 (90%)	3 (10%)
Without prior symptoms	860 (83%)	69 (80%)
Newly developed acute symptoms, median (IQR)	2 (0, 4)	5 (4, 6)
Livability index (mean, SD)	0.05 (0.11)	0.03 (0.11)

The risk of post-acute COVID symptoms at 100 days was lower in vaccinated participants compared with unvaccinated participants, though with wide CIs (adjusted OR (aOR): 0.5; 95% CI: 0.2 to 1.5; adjusted for age, sex, time period and livability index, see table 2). This association was similar in both periods of infection. Vaccination was associated with fewer new symptoms during the acute phase, as a proxy for severity of acute infection (beta: −0.9; 95% CI: −1.6 to –0.3, same adjustments as prior analysis) and was also associated with asymptomatic COVID-19 (aOR: 2.8; 95% CI: 1.6 to 5.1; same adjustments as prior analyses).

**Table 2:** ORs for post-acute COVID symptoms at day 100

Symptoms at 100 days	OR (95% CI)(adjusted 1)	OR (95% CI)(adjusted 2)	OR (95% CI)(adjusted 3)
Vaccination			
In all included participants (86/1128)	0.4 (0.2 to 0.8)	0.5 (0.2 to 1.5)	0.6 (0.1 to 2.5)
In symptomatic participants (86/819)	0.5 (0.2 to 0.9)	0.6 (0.1 to 2.8)	0.7 (0.1 to 3.5)
Number of symptoms in acute phase			
In all included participants (86/1128)	1.5 (1.4 to 1.6)	1.5 (1.3 to 1.6)	–
In symptomatic participants (86/819)	1.4 (1.2 to 1.5)	1.4 (1.2 to 1.5)	–

Adjusted 1): by age; sex and livability index.

Adjusted 2): Aadjustment 1+period of infection.

Adjusted 3): Aadjustment 2+number of new acute symptoms.

In participants with symptoms during the acute phase (n=819), the number of newly developed symptoms in the acute phase was positively associated with new post-acute COVID symptoms at 100 days after a positive test (aOR: 1.4; 95% CI: 1.2 to 1.5 for each additional symptom in the acute phase, see table 2).

Including the number of newly developed symptoms during the acute phase (as a proxy for severity of acute infection), the association between vaccination and post-acute COVID symptoms at 100 days, changed from 0.6 to 0.7, which implies that severity of disease is a possible mediator in the effect of vaccination on post-acute COVID symptoms.

### Determinants associated with post-acute COVID symptom clusters

**Figure 4:**
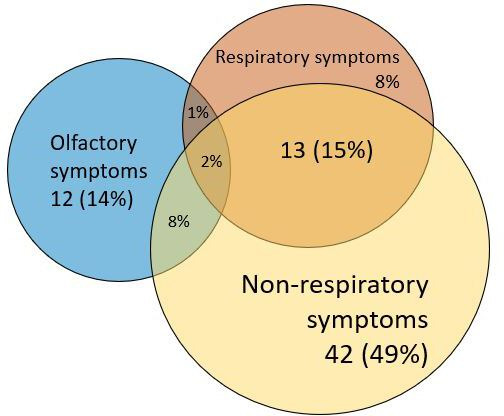
Distribution and overlap of three identified post-acute COVID symptom clusters. Total 86 participants with symptoms at day 100 (7.6%). Two of them were not part of any cluster. Respiratory symptoms: cough, shortness of breath and sore throat. Non-respiratory symptoms: fatigue and headache. Olfactory symptoms: loss of taste or smell. For example: 42 (49%) participants reported only non-respiratory symptoms at day 100 (newly developed at the acute phase).

Within participants with post-acute COVID symptoms at day 100 (n=86), most had only non-respiratory symptoms at day 100 (42 (49%)), followed by only olfactory symptoms (12 (14%)) (see figure 4 and online supplemental table 4). Participants with respiratory symptoms at day 100 (23 (27%)) often reported symptoms from the non-respiratory (ie, headache and fatigue) cluster too (13 (15%)).

Only women reported post-acute COVID olfactory symptoms at day 100. Post-acute COVID respiratory symptoms were observed more in men than women (5 (71%) participants with post-acute COVID respiratory symptoms were men vs 14 (26%) in other symptom clusters) and in those who already reported other symptoms prior to infection (4 (57%) vs 5 (9%) in other symptom clusters reported prior symptoms). Post-acute COVID olfactory and post-acute COVID respiratory symptoms originated more frequently in the first half of 2021 (during the alpha variant) than during the second half of 2021 (during the delta variant) compared with post-acute COVID non-respiratory symptoms (18 (95%) vs 35 (84%) originated after the alpha variant, respectively). Participants with non-respiratory symptoms at day 100 were in many ways similar to patients without symptoms at day 100, for example, concerning vaccination status and period of infection (online supplemental table 4).

Vaccination in the acute phase was not associated with the presence of only post-acute COVID symptoms at day 100 from the non-respiratory cluster (aOR: 1.0; 95% CI: 0.3 to 4.4, see online supplemental table 5). The number of symptoms in the acute phase was associated with the presence of only post-acute COVID symptoms at day 100 of the non-respiratory cluster (aOR: 1.3; 95% CI: 1.1 to 1.5).

### Sensitivity analyses

Sensitivity analyses using different definitions for prior symptoms (25% or 75% of prior observations with symptoms), different definitions for recovery (7 days or 21 days without symptoms), 60 days of follow-up instead of 100 days and including participants without recovery before loss to follow-up (assuming direct recovery or recovery at timepoint) showed small differences in estimates which did not alter conclusions (details reported in online supplemental material: Sensitivity analyses).

## Discussion

Using data from a population-based app, with voluntary users, in the Netherlands, we found that between 5% and 10% of participants still reported symptoms, newly developed during the acute phase, at 100 days after acute COVID-19. Most common post-acute symptoms were loss of smell and taste, fatigue and shortness of breath.

Using a data-driven approach we found three post-acute COVID symptom clusters: non-respiratory symptoms (headache and fatigue); olfactory symptoms; and respiratory symptoms. The symptom clusters differed in the moment that they originated (during the alpha or delta period), frequency, effect of vaccination and characteristics of patients. This suggests that multiple mechanisms play a role in the development of post-acute COVID symptoms.

We found a negative association between vaccination and the persistence of post-acute COVID symptoms, however CIs were wide indicting caution when interpreting these results. With the number of newly developed symptoms in the acute phase as a proxy for severity of disease, it was associated with persistence of post-acute COVID symptoms. Severity of acute disease is a possible mediator in the effect of vaccination in the persistence of symptoms after acute COVID-19. The negative association of vaccination was not seen in participants with only non-respiratory post-acute COVID symptoms, which was half of the participants with post-acute COVID symptoms.

Prior research from app data has shown a similar frequency of post-acute COVID symptoms as in our study, and also reported similar results with regard to the influence of sex and age.^15 16^ Several meta-analyses have also led to incidence estimates of 5%–10% for post-COVID^2 3 9^ The positive association between severity of the acute disease and post-acute COVID symptoms was described in prior research.^17 18^ However, in most studies severe COVID-19 was considered after hospitalisation or admission to the intensive care unit, while in our study, including only community-dwelling patients, severity was measured in more detail. Still the risk for post-acute COVID symptoms at 100 days after infection increased with 40% per additional acute symptom, indicating that the association between severity of acute COVID-19 and post-acute COVID symptoms is also of relevance in outpatients. Antonelli *et al* previously reported associations between vaccination status and fewer symptoms in the acute phase with fewer long-lasting symptoms,^19^ which is in line with our results, and has been confirmed with other data sources and methods.^20 21^ We found a lower prevalence of post-acute COVID symptoms in the part of the research period in which the delta variant was prevalent, which is consistent with prior reports.^22 23^ However, similar to our results, vaccination might have influenced this difference.

In an overview of seven studies on post-acute COVID symptom clusters, most studies clustered symptoms in neurologic, cardiorespiratory or systemic/inflammatory.^24^ In the case of post-COVID, in which several definitions exist, we encourage to extract data from population-based sources and use the duration of symptoms (excluding participants with only acute symptoms) for clustering, without restricting it to a specific timeframe. However, data-driven techniques should always be combined with clinical expertise as also discussed by Hulsen *et al*, to maximise their synergistic potential.^25^

Because the data were collected during the development of the epidemic, we were able to measure newly developed symptoms on the individual level, by taking prior symptoms into account. Another strength of this study is that participants were community dwelling instead of selected from hospitals or from other healthcare resources. In addition, data from the app were detailed, that is, about symptomatology in the prior, acute and post-acute infection phase, and about many variables that allowed adjustment for confounding. We used several assumptions and definitions in our analyses, which did not appear crucial to the main results, as shown in the sensitivity analyses.

This study has several limitations. The participants were self-selected app users, that is, they started, paused and stopped the usage of the app voluntarily. This has resulted in oversampling of relatively older people (taking the time or considered it more important to participate) and oversampling of users experiencing symptoms. It is also possible that people with very severe (post-acute COVID) symptoms will be less likely to use the app. Because it is likely that participants with (post-acute COVID) symptoms will fill in the app for a longer period of time, we did not censor on duration of usage before 100 days and we performed a sensitivity analyses including participants who were lost to follow-up assuming immediate recovery or recovery at 100 days. Assuming recovery at 100 days in all participants who were lost to follow-up yielded higher estimates of post-acute COVID symptoms at day 100 (up to 30%), but this scenario is unlikely, since we believe that most of these participants stopped using the app because they did not experience symptoms anymore. Several post-acute COVID symptoms currently known (such as brain fog and depression) were not part of our survey and hence could not be used in our analyses. Further, the presence of multicollinearity arose due to the temporal concurrence of various SARS-CoV-2 variants and vaccination, in which vaccination and the variants were highly correlated (few participants were vaccinated during the period of the alpha variant and few unvaccinated during the period of the delta variant). Lastly we did not have details about comorbidities or body mass index; and all data were self-reported with possible measurement error due to misinterpretation. As these factors influence the probability of development of severe COVID-19, they might be of relevance for targeting (preventive) therapies for post-COVID.

In conclusion, in continuous data from the general population the incidence of post-acute COVID symptoms is between 5% and 10% and half of these patients suffer non-respiratory symptoms. A more severe acute infection is associated with a higher probability of prolonged post-acute COVID symptoms. In addition to the preventive effect of developing COVID-19, vaccination was associated with less post-acute COVID symptoms, but not with less post-acute COVID non-respiratory symptoms. Since evidence on aetiology of post-COVID still needs to be built up, our findings might help to find subgroups at risk for developing specific kinds of post-COVID for which eventually targeted interventions might become available.

## supplementary material

10.1136/bmjopen-2024-087235 online supplemental file 1

## Data Availability

Data are available in a public, open access repository.
