# Supplementary material for: Incidence, symptom clusters and determinants of post-acute COVID symptoms: a population-based surveillance in community-dwelling users of the COVID RADAR app
Source: BMJ Open. 2024 Sep 10;14(9):e087235. doi: 10.1136/bmjopen-2024-087235 (PMC11409358; doi:10.1136/bmjopen-2024-087235)
Supplement: online supplemental file 1 [file bmjopen-14-9-s001.pdf]

Incidence, symptom clusters and determinants of post-acute COVID symptoms- a population based surveillance in community dwelling users of the COVID RADAR app.

Supplemental Material

Outline

Supplemental Table 1: Overview relevant variables in the app 3

Supplemental Figure 1: Visualization of phases surrounding report of positive SARS-CoV-2 test.......... 4

Supplemental Figure 2: Directed Acyclic Graph (DAG) of association between vaccination and persistence of post-acute COVID symptoms 4

Methods (details).......... 5

Supplemental Table 2: Included and excluded participants; demographics and app use 7

Supplemental Figure 3: Patterns of app usage.......... 8

Supplemental Table 3: Incidence and median duration of symptoms in days (N=1,478).......... 9

Supplemental Figure 4: Correlation heatmap sensitivity analysis (using participants without recovery before follow-up, assuming recovery at end of follow-up) 9

Supplemental Table 4: Characteristics of infected participants in the acute phase; stratified by symptom cluster 10

Supplemental Table 5: Association between vaccination, number of symptoms during the acute phase and having only Non-respiratory post-acute COVID symptoms (Fatigue and Headache) at days 100 11

Sensitivity analyses 12

References 14

Supplemental Table 1: Overview relevant variables in the app

**Variable name**	**Variable type**	**Question**
Gender	categorical (M/F/O/N)	What is your gender? (male, female, other, none)
Age	<18, 19-39, 40-59, 60+	What is your age?
Cough	binary	Did you cough? (no/yes)
Soar Throat	binary	Did you have a sore throat? (no/yes)
Fever	binary	Did you have a fever? (no/yes)
Shortness of Breath	binary	Did you have shortness of breath? (no/yes)
Bowel symptoms	binary	Did you have stomach issues? (no/yes)
Chest Pain	binary	Did you have pain in the chest? (no/yes)
Eye problems	binary	Did you have swollen eyes? (no/yes)
Loss of smell/taste	binary	Did you have loss of smell or taste? (no/yes)
Fatigue	categorical (N, Y1, Y2)	Did you have Fatigue? (no, yes mild, yes extreme)
Headache	binary	Did you have a headache? (yes/no)
COVID test	categorical (N, Y1, Y2)	Were you tested for COVID-19? (no/yes - in the past 2 weeks/yes - more than 2 weeks ago)
Vaccination	Binary	Did you receive all SARS-CoV-2 vaccinations?
**Other external data**		
Livability index	Continuous	Z-score for livability of postal code
pc	ID variable	What is your postal code? (four digits)
date	timestamp	date user answered questionnaire

Supplemental Figure 1: Visualization of phases surrounding report of positive SARS-CoV-2 test

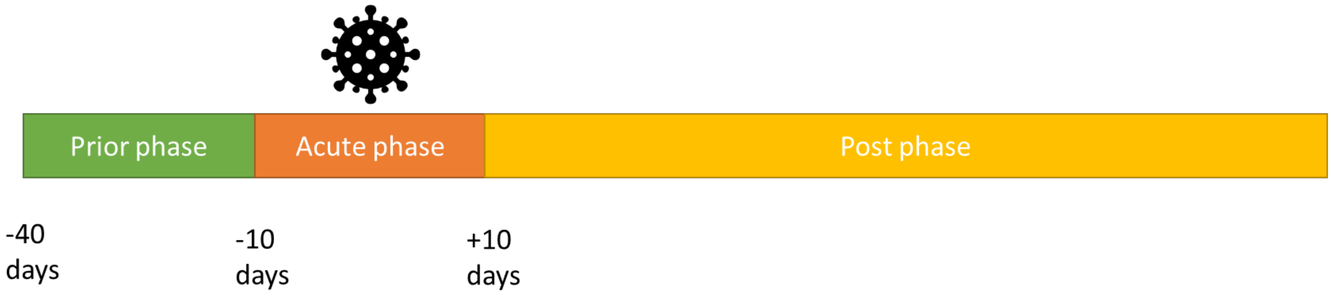

Supplemental Figure 2: Directed Acyclic Graph (DAG) of association between vaccination and persistence of post-acute COVID symptoms

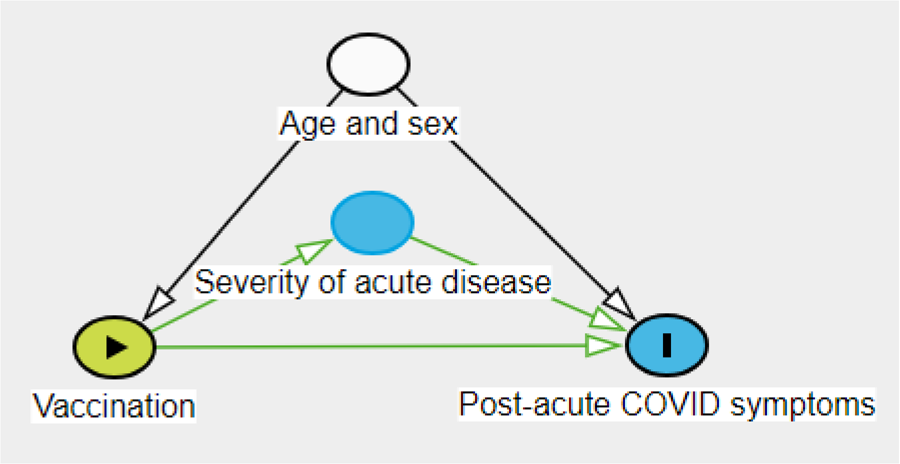

The factors age and sex are confounders of the association between vaccination and post-acute COVID symptoms. The effect of vaccination on post-acute COVID symptoms is both direct and in-direct via the severity of acute disease. Severity of acute disease is therefore a *mediator.*

Methods (details)

COVID RADAR app

The primary aim of the app was to organize a population-based surveillance of the development of COVID-19 over time. Different national (social) media campaigns encouraging usage of the app resulted in 284,000 individual users that filled out the questionnaire more than 8.5 million times between April 2020 and February 2022. The questionnaire was dynamic, which allowed for updating questions in response to changes, for instance, changes in mitigation measures and scientific insights related to the pandemic.

Statistical analyses

For binary variables percentages were reported. For continuous variables mean or medians were reported, with standard deviations (SD), 95%- confidence intervals (CI) or interquartile range (IQR). We used bar graphs to describe patterns of app use surrounding a positive test. Participants answered yes/no questions about 10 symptoms. The symptom fatigue was categorical: “no”, “mild” or “extreme fatigue”. From this symptom variable two dummy variables were made resulting in 11 unique symptom variables.

Clustering method and data selection

For this analysis data of participants were used when at least two symptoms were present for at least 15 days, to confirm they lasted longer than the acute phase. Because few participants reported symptoms ‘Fever’, ‘Eye symptoms’, ‘Bowel symptoms’ and ‘Chest pain’ for over 15 days, these symptoms were excluded from the correlation heatmap. The symptoms mild and extreme fatigue are mutually exclusive. Therefore we used the maximum duration of one of these symptom. Spearman correlation coefficients were computed and mapped.

Multivariate analyses

Age was categorized in four groups: 0-18 years, 19-39 years, 40-59 years, and over 60 years, with the modus (>60 years) as reference category (this categorization was by design of the app). Sex was categorized in male or female, with male as reference category. A participant was considered vaccinated when the participant reported complete vaccination at the time of their positive SARS-CoV-2 test result. When vaccination status was unknown at the time of their infection, this was because vaccines were not availability yet, and therefore we assumed that the participant was unvaccinated when this infection occurred during the period when no vaccine was available yet. Because prior research indicated that COVID-19 and post-acute COVID symptoms are influenced by regional factors such as the social and living environment, we also included the livability index.[1, 2] This is a score (normalized to a Z-score) for each postal code, derived from over 100 variables about social, economic, and physical factors in a particular region.[3]

During the analysis period (November 2020 until November 2021) two variants of SARS-CoV-2 were prevalent (alpha variant most prevalent until June 2021 and delta variant most prevalent from July 2021). Given symptom profiles varied with SARS-CoV-2 variants, this can be a possible confounder in the association between vaccination and persistence of symptoms.[4] To adjust for this confounding we split the research period into two parts of six months each (December 2020 until June 2021 (alpha variant); July 2021 until November 2021 (delta variant) and added this period as a variable to the multivariate regression model.[5]

Sensitivity analyses

The definitions of a 'prior symptom' (symptom reported at >50% of a participant's available observations during the prior phase) and recovery (no symptom in the 14 consecutive days) were based on clinical judgement and not on prior literature. In addition the definition of recovery was in line with the maximum of 14 days gap in app usage in the inclusion criteria. Because these definitions may have influenced the estimate of participants with prolonged symptoms we performed sensitivity analyses considering a symptom a 'prior symptom' when this symptoms was reported in over 75% or 25% of the available reports in the prior phase and using 7 and 21 days for the definition of recovery. These sensitivity analyses also alter the inclusion criteria; the participants were allowed to have a 7 or 21 days gap in app usage before being considered lost to follow-up. This results in different estimations of incidence of 'new post-acute COVID symptoms' at 100 days. In addition to the analyses of recovery in the first 100 days, we analysed recovery from newly developed symptoms at 60 days (similar to the WHO definition).

The group without recovery before loss to follow-up and a follow-up of less than 100 days was excluded. We performed two sub-analyses for research question c (associations between factors in the acute phase and persistence of symptoms until 100 days and the possible mediation of severity of acute disease in the association between vaccination and post-acute COVID symptoms) in which we included these participants. In the first we assumed that they recovered at day of loss to follow-up; in the second we assumed that they did not recover before 100 days of follow-up. For research question b) (symptom cluster) we performed one sub-analysis using participants without recovery before loss to follow-up, assuming recovery at day of loss of follow-up.

Supplemental Table 2: Included and excluded participants; demographics and app use

		Included	Excluded
**N**		1478	2164
**Sex**	Female	865 (58.5%)	1284 (59.3%)
**Age**	<18	61 (4.1%)	130 (6.0%)
	19-39	78 (5.3%)	287 (13.3%)
	40-59	480 (32.5%)	1038 (48.0%)
	>60	859 (58.1%)	709 (32.8%)
**Vaccinated before infection**		614 (41.5%)	1081 (50.0%)
**Observations in prior phase per patients (median; IQR)**		10 (6, 21)	1 (0, 2)
**Without prior symptoms**		1216 (82.3%)	2039 (94.2%)
**Newly developed symptoms during acute phase, median (IQR)**		2 (0, 4)	4 (2, 5)
**Days of non-censored app use after test, median (IQR)**		82 (30 , 173)	8 (0 , 26)

(IQR: interquartile range)

Supplemental Figure 3: Patterns of app usage

a) Number of unique users each block of 10 days surrounding positive test

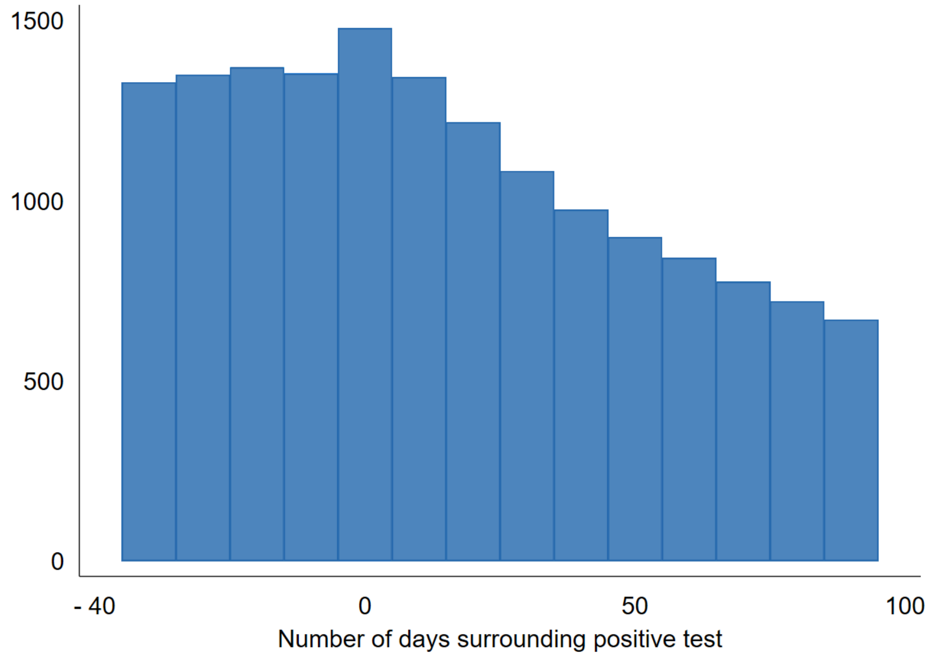

b) Number of observations each block of 10 days surrounding positive test

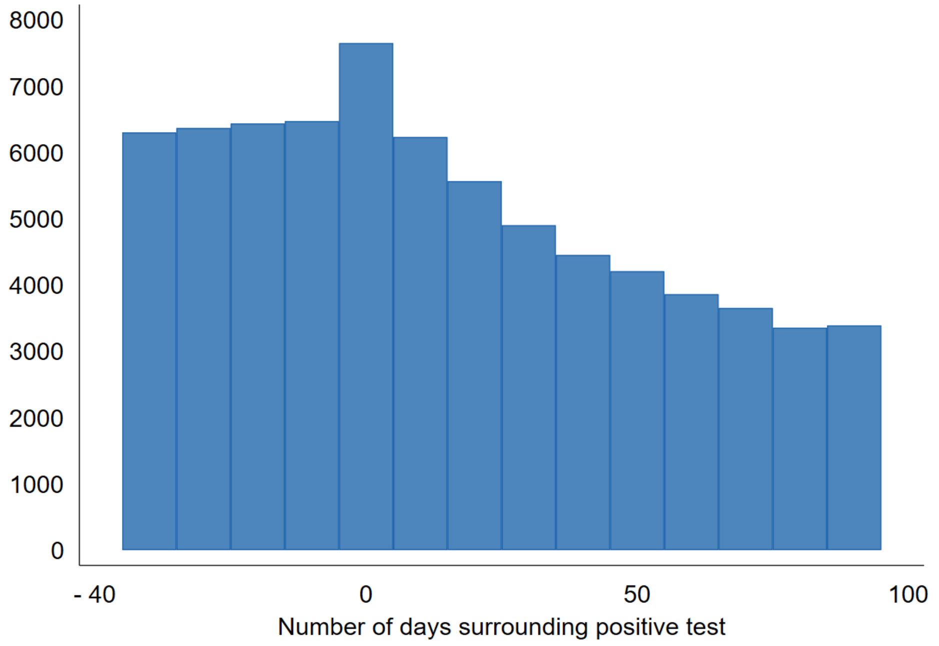

Supplemental Table 3: Incidence and median duration of symptoms in days (N=1,478)

Symptom	Frequency	Proportion	Median duration in days (IQR)
**Cough**	776	53%	10 (6, 18)
**Sore Throat**	500	34%	6 (4, 10)
**Fever**	352	24%	5 (3, 9)
**Shortness of Breath**	202	14%	9 (5, 21)
**Gastro-Intestinal**	240	16%	5 (3, 10)
**Chest Pain**	130	9%	7 (4, 12)
**Swollen eyes**	71	5%	7 (5, 12)
**Loss of smell/taste**	347	23%	13 (7, 24)
**Mild Fatigue**	653	44%	14 (7, 30)
**Extreme Fatigue**	168	11%	9 (5, 20)
**Headache**	589	40%	7 (4, 13)

IQR: interquartile range

Supplemental Figure 4: Correlation heatmap sensitivity analysis (using participants without recovery before follow-up, assuming recovery at end of follow-up)

Spearman correlations of associations between duration of symptoms in participant with at least two symptoms lasting for over 15 days.

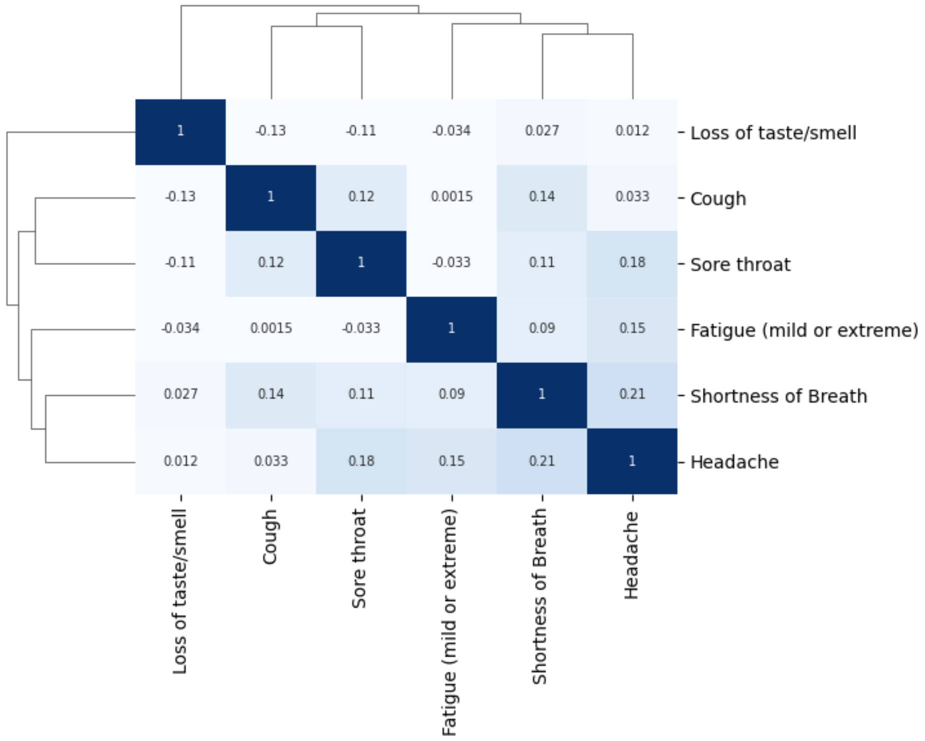

Supplemental Table 4: Characteristics of infected participants in the acute phase; stratified by symptom cluster

without post-acute COVID symptoms at day 100 or post-acute COVID symptoms from one of the three symptom clusters; excluding participants with symptoms from multiple clusters (n=23; 27%)

**Symptoms at 100 days**		**No**	**Non-respiratory**	**Olfactory**	**Respiratory**
**Number**		1042	42	12	7
**Sex**	Female	603 (58%)	28 (67%)	12 (100%)	2 (29%)
**Age**	<18	45 (4%)	0 (0.0%)	1 (8%)	1 (14%)
	19-39	55 (5%)	4 (10%)	1 (8%)	0 (0%)
	40-59	351 (34%)	18 (43%)	7 (58%)	4 (58%)
	>60	591 (57%)	20 (48%)	3 (25%)	2 (29%)
**Vaccinated at moment of infection**		263 (25%)	8 (19%)	0 (0%)	0 (0.0%)
**Without prior symptoms**		860 (83%)	38 (91%)	11 (92%)	3 (43%)
**Newly developed acute symptoms, median (IQR)**		2 (0, 4)	5 (3, 6)	6 (4, 6.5)	4 (4, 4)
**Livability index (Z-score, mean, SD)**		0.05 (0.11)	0.03 (0.1)	0.02 (0.11)	0.01 (0.11)
**Time of infection**	Period 1 (Nov 20 – Jun 21)	787 (75%)	35 (84%)	11 (92%)	7 (100%)
	Period 2 (Jul 21 – Nov 21)	255 (25%)	7 (16%)	1 (8%)	0 (0%)

Supplemental Table 5: Association between vaccination, number of symptoms during the acute phase and having only Non-respiratory post-acute COVID symptoms (Fatigue and Headache) at days 100

(clusters with olfactory or respiratory symptoms were omitted because of non-positivity in vaccination)

Non respiratory post-acute COVID symptoms at 100 days	Odds ratio (95% CI) (adjusted 1)	Odds ratio (95% CI) (adjusted 2)	Odds ratio (95% CI) (adjusted 3)
**Vaccination**			
**In all included participants (42/1084)**	0.7 (0.3 -1.5)	1.0 (0.3 – 4.4)	2.0 (0.4 - 10)
**In symptomatic participants (42/775)**	0.8 (0.4 – 1.8)	2.8 (0.5 – 17)	3.7 (0.6 – 25)
**Number of symptoms**			
**In all included participants (42/1084)**	1.4 (1.2 – 1.6)	1.4 (1.2 – 1.6)	-
**In symptomatic participants (42/775)**	1.3 (1.1 – 1.5)	1.3 (1.1 – 1.5)	

Adjusted 1) by age; sex and livability index

Adjusted 2) Adjusted 1 + period of infection

Adjusted 3) Adjusted 2 + number of new acute symptoms

Sensitivity analyses

A) Overview of participants with post-acute COVID symptoms at day 100, using different definitions of prior symptoms and time of recovery

	Prior symptom: 25% of prior observations with symptom			Prior symptom: 50% of prior observations with symptom			Prior symptom: 75% of prior observations with symptom		
	Recovery definition 7 days	Recovery definition 14 days	Recovery definition 21 days	Recovery definition 7 days	**Recovery definition 14 days**	Recovery definition 21 days	Recovery definition 7 days	Recovery definition 14 days	Recovery definition 21 days
**Primary analysis** Included	1055	1144	1181	1040	1128	1167	1028	1117	1156
**Primary analysis** Symptoms at 100 days	54 (5.1%)	78 (6.8%)	95 (8.0%)	56 (5.4%)	86 (7.6%)	105 (9.0%)	57 (5.5%)	91 (8.2%)	114 (9.9%)
**Secondary analysis** Included	1389	1389	1389	1389	1389	1389	1389	1389	1389
**Secondary analysis** Symptoms at 100 days maximum	388 (28%)	323 (23%)	303 (22%)	405 (29%)	347 (25%)	327 (24%)	418 (30%)	363 (26%)	347 (25%)
Symptoms at 100 days minimum	54 (3.9%)	79 (5.7%)	95 (6.8%)	56 (4.0%)	87 (6.3%)	105 (7.6%)	57 (4.1%)	92 (6.6%)	114 (8.2%)

B) Overview of participants with post-acute COVID symptoms at day 60, using different definitions of prior symptoms and time of recovery

	Prior symptom: 25% of prior observations with symptom			Prior symptom: 50% of prior observations with symptom			Prior symptom: 75% of prior observations with symptom		
	Recovery definition 7 days	Recovery definition 14 days	Recovery definition 21 days	Recovery definition 7 days	**Recovery definition 14 days**	Recovery definition 21 days	Recovery definition 7 days	Recovery definition 14 days	Recovery definition 21 days
**Primary analysis** Included	1194	1286	1326	1176	1268	1309	1161	1254	1295
**Primary analysis** Symptoms at 60 days	79 (6.6%)	117 (9.1%)	155 (11.7%)	83 (7.1%)	131 (10.3%)	169 (12.9%)	87 (7.5%)	139 (11.1%)	180 (13.9%)
**Secondary analysis** Included	1621	1621	1621	1621	1621	1621	1621	1621	1621
**Secondary analysis** Symptoms at 60 days maximum	506 (31%)	452 (27.9%)	450 (28%)	528 (33%)	484 (30%)	481 (30%)	547 (34%)	506 (31%)	506 (31%)
Symptoms at 60 days minimum	103 (6.4%)	147 (9.1%)	202 (12.5%)	110 (6.8%)	165 (10.1%)	219 (13.5%)	115 (7.1%)	177 (10.9%)	234 (14.4%)

C) Association between vaccination, number of symptoms during the acute phase and having post-acute COVID symptoms at days 100 including participants loss to follow-up before recovery

Symptoms at 100 days	Odds ratio (95% CI) (adjusted 1)	Odds ratio (95% CI) (adjusted 2)	Odds ratio (95% CI) (adjusted 3)
**Vaccination**			
**Assuming direct recovery (87/1389)**	0.4 (0.2 – 0.9)	0.5 (0.2 -1.5)	0.6 (0.1 – 2.1)
**Assuming recovery at day 100 (347/1389)**	0.8 (0.6 – 1.1)	0.8 (0.4 -1.3)	0.9 (0.4 – 1.8)
**Number of symptoms (in symptomatic patients)**			
**Assuming direct recovery (87/1080)**	1.3 (1.2 – 1.4)	1.3 (1.2 – 1.4)	-
**Assuming recovery at day 100 (347/1080)**	1.3 (1.2 – 1.4)	1.3 (1.2 - 1.4)	-

Adjusted 1) by age; sex and livability index
Adjusted 2) Adjusted 1 + period of infection
Adjusted 3) Adjusted 2 + number of new acute symptoms

D) Association between vaccination, number of symptoms during the acute phase and post-acute COVID symptoms at days 60

Symptoms at 100 days	Odds ratio (95% CI) (adjusted 1)	Odds ratio (95% CI) (adjusted 2)	Odds ratio (95% CI) (adjusted 3)
**Vaccination**			
**In all included participants (131/1268)**	0.5 (0.3 – 0.8)	0.6 (0.3 – 1.5)	0.8 (0.2 – 2.4)
**In symptomatic participants (131/924)**	0.5 (0.3 – 0.8)	0.9 (0.3 – 3.0)	1.0 (0.3 – 3.5)
**Number of symptoms**			-
**In all included participants (131/1268)**	1.5 (1.4 – 1.6)	1.5 (1.5 – 1.6)	
**In symptomatic participants (131/924)**	1.4 (1.3 – 1.5)	1.4 (1.2 – 1.5)	

Adjusted 1) by age; sex and livability index
Adjusted 2) Adjusted 1 + period of infection
Adjusted 3) Adjusted 2 + number of new acute symptoms

References

1. Kong, J.D., E.W. Tekwa, and S.A. Gignoux-Wolfsohn, *Social, economic, and environmental factors influencing the basic reproduction number of COVID-19 across countries.* PloS one, 2021. **16**(6): p. e0252373.
2. König, B.H., et al., *Prognostic factors for persistent fatigue after COVID-19: a prospective matched cohort study in primary care.* British Journal of General Practice, 2023. **73**(730): p. e340-e347.
3. Zaken, M.v.B., *Leefbarometer 3.0*. 2022, Ministerie van Binnenlandse Zaken: https://data.overheid.nl/dataset/leefbaarometer-meting-2020.
4. Fernández-de-Las-Peñas, C., et al., *Associated-onset symptoms and post-COVID-19 symptoms in hospitalized COVID-19 survivors infected with Wuhan, Alpha or Delta SARS-CoV-2 variant.* Pathogens, 2022. **11**(7): p. 725.
5. Geubbels, E., et al., *The daily updated Dutch national database on COVID-19 epidemiology, vaccination and sewage surveillance.* Scientific Data, 2023. **10**(1): p. 469.
