# Supplementary material for: Incidence, symptom clusters and determinants of post-acute COVID symptoms: a population-based surveillance in community-dwelling users of the COVID RADAR app
Source: BMJ Open. 2024 Sep 10;14(9):e087235. doi: 10.1136/bmjopen-2024-087235 (PMC11409358; doi:10.1136/bmjopen-2024-087235)
Supplement: Supplementary file 2 [file bmjopen-2024-087235.reviewer_comments.pdf]

PEER REVIEW HISTORY

BMJ Open publishes all reviews undertaken for accepted manuscripts. Reviewers are asked to complete a checklist review form and are provided with free text boxes to elaborate on their assessment. These free text comments are reproduced below.

ARTICLE DETAILS

**TITLE (PROVISIONAL)**	Incidence, symptom clusters and determinants of post-acute COVID symptoms - a population based surveillance in community dwelling users of the COVID RADAR app.
**AUTHORS**	van Dijk, Willian; Haaksma, Miriam; Mook-Kanamori, Dennis O.; Visser, Leo; Numans, Mattijs; van Hylckama Vlieg, A; Rosendaal, Frits; Kiefte-de Jong, Jessica

VERSION 1 - REVIEW

**REVIEWER NAME**	Sayeed, Muneeba Ahsan
**REVIEWER AFFILIATION**	Sindh Infectious Diseases Hospital & Research Center
**REVIEWER CONFLICT OF INTEREST**	None
**DATE REVIEW RETURNED**	13-May-2024

**GENERAL COMMENTS**	The available data on Long COVID is limited, therefore, the relevance of this topic persists.<br>Few suggestions<br>Abstract: Please rephrase it and make it clearer and crisper<br>Introduction section: Line 84-92: Rephrase. Mention these questions as objectives and write these in continuous sentence format like 'In this study, our objectives were to evaluate the symptoms of COVID during the acute phase and determine their duration, to identify symptoms persisting beyond the acute phase and explore their associations, and to investigate further aspects such as the factors causing its persistence until 100 days and its relationship with vaccination status.'<br>Methods Section<br>Line 97: Remove this line "A detailed overview of the COVID RADAR app has been published elsewhere". Instead mention about this app here. Other information related to this app is mentioned in supplementary data. I would suggest you mention all the relevant details about this app here.<br>Results Section:<br>Line 166: 58.000 participants used this app. Please mention how this number was calculated. Is this 58,000? Additionally, the number of included participants was 1,478; however, in line 186, it is incorrectly stated as 1.478.<br>Line 168: is it 1,675 or 1.675?<br>Line 171: instead of "was" write "were"<br>Line 177 onwards: In this section mention both 'N (number)' and '% (percentage)'. Follow same format<br>throughout.

	Table 1: In first row mention percentage for total number. Calculate p-value to see the statistical significance.<br>Discussion Section:<br>Line 276: Please elaborate the findings of other studies

**REVIEWER NAME**	Sharath, Sherene E.
**REVIEWER AFFILIATION**	SUNY Downstate Health Sciences University, Department of Surgery
**REVIEWER CONFLICT OF INTEREST**	None
**DATE REVIEW RETURNED**	15-May-2024

**GENERAL COMMENTS**	I congratulate the authors on a very thoughtful study with robust sample size and unique in its ability to provide insight on a tough-to-study subject. The topic is of importance and the study design is appropriate to the descriptive (prevalence of post-COVID symptoms) and associative (risks dependent on vaccination) questions. The phrasing and syntax, on occasion, makes it challenging to appreciate the true intent of the study. This is meaningfully interruptive to interpretation as there are a number of unconventional or newly defined variables. I've provided key suggestions where major revision is needed but would recommend revising and clarifying further.<br><br>Syntax note: The manuscript is well-written but there are certain grammatical conventions and syntax structures that impede continuity. For example, introduction (question a) "What are "the" symptoms started..." Similarly question b may be better phrased as an objective - "To identify symptoms that lasted longer than the acute phase..." This direct application of this question is challenging to understand. Please also review and revise for appropriate punctuation.<br><br>Included below are a few suggestions to increase the reproducibility of the study.<br><br>Abstract:<br>- How were prolonged/persistent symptoms defined (i.e., what is the distinction between the acute and prolonged phase of the infection?) This is critical to understanding the significance of findings in the abstract.<br><br>Introduction:<br>- Recommend identifying one primary research question to refine the study objective and classify all others as secondary questions.<br>- In this vein, would recommend revising these questions to objectives.<br>- Purpose or meaning of question b is unclear (see comment above in syntax)<br>- Purpose or meaning of question c is not immediately clear. I sense this is referring to risk factors. How are the acute and prolonged phases defined? Persistence is defined as symptoms >100 days?<br>- Please rephrase question d - this is substantially challenging to parse. I see that exposure = vaccination and mediator = acute disease severity but what is the outcome?<br><br>Methods:<br>- Typo for "symptom" on line 113

	- The definitions for duration and symptoms seem robust. Are these based on any previous work or are these entirely defined by the study?<br>- Would consider simplifying lines 121-126. This is a bit hard to follow. Would recommend including Supplemental Figure 2 in the main manuscript, if possible.<br>- Would recommend rephrasing lines 137-141. Perhaps along the lines of..."Correlation matrix heat maps were used to delineate new symptoms that developed during the acute COVID-19 phase and lasted longer than the acute phase" (is this the prolonged phase)? Would recommend remaining consistent with the definitions previously identified under the definitions section.<br>- Lines 151-156: I do not believe that what is being described here is a mediation analysis. The exposure (vaccination) and outcome (# of newly developed symptoms) are being assessed but what is the third variable (i.e., mediator)? Reading on, I realize that the exposure (vaccination) and outcome (prolonged symptoms) are being examined in the presence of severity. Would suggest revising this section to be clearer as this is not evident without substantial consideration. In addition, simply adding or removing the variable associated with severity may not equate to a mediation analysis. This sounds to me more like a sensitivity analysis. Would recommend reconsidering methodology.<br><br>Results:<br>- The description of the eligible cohort is exceptional. Very elegant. It is particularly helpful to understand the characteristics of those who were excluded.<br>- The subheadings are very clear - would recommend simplifying the research questions in the introduction to objectives that mirror these subheadings.<br>- Table 1: suggest replacing "moment" with "time"<br><br>Discussion:<br>- Well considered. Limitations are appropriately oriented in strengths.

VERSION 1 – AUTHOR RESPONSE

Reviewer: 1

Dr. Muneeba Ahsan Sayeed, Sindh Infectious Diseases Hospital & Research Center

Comments to the Author:

The available data on Long COVID is limited, therefore, the relevance of this topic persists.

Few suggestions

Abstract: Please rephrase it and make it clearer and crisper

Thank you for the careful review of our manuscript. We rephrased the abstract and shortened it, to make it more clear. Because this was an extensive change, we refer to the track-change document.

Introduction section: Line 84-92: Rephrase. Mention these questions as objectives and write these in continuous sentence format like 'In this study, our objectives were to evaluate the symptoms of COVID during the acute phase and determine their duration, to identify symptoms persisting beyond the acute phase and explore their associations, and to investigate further aspects such as the factors causing its persistence until 100 days and its relationship with vaccination status.'

We thank the reviewer for this suggestion. We changed this part:

Line 94: In this study, our main objective was to identify symptoms persisting beyond the acute phase (post-acute COVID symptoms). Secondary, we aimed to identify clusters of post-acute COVID symptoms, i.e. possible subtypes of Post-COVID, and we investigated which factors in the acute phase (such as severity of disease and vaccination status) were associated with (clusters of) symptoms persisting at least 100 days after a positive test.

Methods Section

Line 97: Remove this line "A detailed overview of the COVID RADAR app has been published elsewhere". Instead mention about this app here. Other information related to this app is mentioned in supplementary data. I would suggest you mention all the relevant details about this app here.

Even though we referred to a previous publication for details of the app, all relevant details were provided in the supplemental material of the current manuscript. We agree that it is much more clear to provide relevant details of the app in the main document; therefore we moved several parts from the supplements to the main document. However, some details are very extensive and would lead to a long description in the main text and potentially a less clear manuscript. Therefore, for the more extensive details (such as the list of questions and sensitivity analyses) we chose to still refer to the supplements.

Results Section:

Line 166: 58.000 participants used this app. Please mention how this number was calculated. Is this 58,000?

Additionally, the number of included participants was 1,478; however, in line 186, it is incorrectly stated as 1.478.

Line 168: is it 1,675 or 1.675?

Apologies. We corrected the numbers. We also included supplementary figure 2 (flowchart about inclusion) into the main document for further clarification of the study population.

Line 171: instead of "was" write "were"

This is corrected.

Line 177 onwards: In this section mention both 'N (number)' and '% (percentage)'. Follow same format throughout.

Thank you for the suggestion. Accordingly, we changed this throughout the text and also in supplemental table 4

Table 1: In first row mention percentage for total number. Calculate p-value to see the statistical significance.

We added the percentage (both 100%) for the total numbers.

In line with our comments to the editors, we want to refrain from p-values. The aim of this table is descriptive. Including p-values would result in erroneously concluding no differences in these small groups. [1-3] Providing these percentages is, in our opinion, the most transparent en clear description of these groups. Therefore, we believe there is no added value of providing the p-value to this descriptive table.

Discussion Section:

Line 276: Please elaborate the findings of other studies

We added several references to prior studies. For example:

Line 343: The positive association between severity of the acute disease and post-acute COVID symptoms was described in prior research. However, in most studies severe COVID-19 was considered after hospitalisation or admission to the Intensive Care Unit, while in our study, including only community dwelling patients, severity was measured in more detail. Still the risk for post-acute COVID symptoms at 100 days after infection increased with 40% per additional acute symptom, indicating that the association between severity of acute COVID-19 and post-acute COVID symptoms is also of relevance in outpatients.

Reviewer: 2

Dr. Sherene E. Sharath, SUNY Downstate Health Sciences University, VA New York Harbor Healthcare System

Comments to the Author:

I congratulate the authors on a very thoughtful study with robust sample size and unique in its ability to provide insight on a tough-to-study subject. The topic is of importance and the study design is appropriate to the descriptive (prevalence of post-COVID symptoms) and associative (risks dependent on vaccination) questions. The phrasing and syntax, on occasion, makes it challenging to appreciate the true intent of the study. This is meaningfully interruptive to interpretation as there are a number of unconventional or newly defined variables. I've provided key suggestions where major revision is needed but would recommend revising and clarifying further.

Syntax note: The manuscript is well-written but there are certain grammatical conventions and syntax structures that impede continuity. For example, introduction (question a) "What are "the" symptoms

started..." Similarly question b may be better phrased as an objective - "To identify symptoms that lasted longer than the acute phase..." This direct application of this question is challenging to understand. Please also review and revise for appropriate punctuation.

We thank the reviewer for the compliments and agree that the manuscript may benefit from clarification. One key part is the continuity of used definitions. We therefore included the term ‘post-acute COVID symptom’, instead of prolonged or long lasting symptom. We also revised several parts of the manuscript, further explained below as responses to you suggestions, to make is more clear. However changes were not limited to these responses. We included several figures (for example the flow diagram and the DAG) which also helps understanding the inclusion/exclusion process.

Line 144: If this duration lasted longer than the ‘acute phase’, this symptoms was considered a ‘post-acute COVID symptom’.

Included below are a few suggestions to increase the reproducibility of the study.

Abstract:

- How were prolonged/persistent symptoms defined (i.e., what is the distinction between the acute and prolonged phase of the infection?) This is critical to understanding the significance of findings in the abstract.

We changed ‘prolonged/persistent symptoms’ into post-acute COVID symptoms throughout the manuscript. We added to the abstract the definition of the ‘acute phase’ and to the methods a definition of post-acute COVID symptoms.

Line 33: Incidence and duration of 10 new symptoms that developed during acute infection, defined as ten days prior and ten days after positive test

Line 144: If this duration lasted longer than the ‘acute phase’, this symptoms was considered a ‘post-acute COVID symptom’.

Introduction:

- Recommend identifying one primary research question to refine the study objective and classify all others as secondary questions.

- In this vein, would recommend revising these questions to objectives.

- Purpose or meaning of question b is unclear (see comment above in syntax)

- Purpose or meaning of question c is not immediately clear. I sense this is referring to risk factors. How are the acute and prolonged phases defined? Persistence is defined as symptoms >100 days?

- Please rephrase question d - this is substantially challenging to parse. I see that exposure = vaccination and mediator = acute disease severity but what is the outcome?

We changed this part (also after feedback from Reviewer 1). The main aim is identifying the persisting of symptoms after the acute phase. To make it more clear, we added the purpose of each aim and we removed question d, which now is included in the last aim. More detailed explanation will follow in the method section, where we included a reference to a DAG in the supplements.

Line 94: In this study, our main objective was to identify symptoms persisting beyond the acute phase (post-acute COVID symptoms). Secondary, we aimed to identify clusters of post-acute COVID symptoms, i.e. possible subtypes of Post-COVID, and we investigated which factors in the acute phase (such as severity of disease and vaccination status) were associated with (clusters of) symptoms persisting at least 100 days after a positive test.

Methods:

- Typo for "symptom" on line 113

Apologies, we corrected this.

- The definitions for duration and symptoms seem robust. Are these based on any previous work or are these entirely defined by the study?

These are not defined based on previous work. We performed several sensitivity analyses, indeed proving robustness of the data. We added to the methods the rationale for these sensitivity analyses.

Line 206: The definitions of a 'prior symptom' (symptom reported at >50% of a participant's available observations during the prior phase) and recovery (no symptom in the 14 consecutive days) were based on clinical judgement and not on prior literature. We performed several sensitivity analyses..

- Would consider simplifying lines 121-126. This is a bit hard to follow. Would recommend including Supplemental Figure 2 in the main manuscript, if possible.

We included supplemental figure 2 in the main document. We simplified the text, into:

Line 148: We included participants who reported their first positive test and had at least three prior app entries. They needed to answer the questionnaire for at least 100 days after their positive test or until they fully recovered from all new symptoms within those 100 days. Participants were considered lost to follow-up if they did not report for 14 days. We excluded those who were lost to follow-up before 100 days after positive test and were not recovered at their last report in the app.

- Would recommend rephrasing lines 137-141. Perhaps along the lines of..."Correlation matrix heat maps were used to delineate new symptoms that developed during the acute COVID-19 phase and lasted longer than the acute phase" (is this the prolonged phase)? Would recommend remaining consistent with the definitions previously identified under the definitions section.

We think this section benefits from using the term 'post-acute COVID symptoms' throughout the manuscript instead of 'prolonged phase'. In addition we clarified we clustered only the post-acute COVID symptoms, by including a part from the supplementary material in the main document.

Line 169: A correlation matrix was used to analyse which new post-acute COVID symptoms were associated with each other (e.g., symptom clusters). Correlations were based on the durations until recovery of each new symptom and clustered these correlations using agglomerative hierarchical clustering, with a complete linkage method. For this analysis data of participants were used when at least two symptoms were present for at least 15 days, to confirm they had symptoms lasting longer than the acute phase.

- Lines 151-156: I do not believe that what is being described here is a mediation analysis. The exposure (vaccination) and outcome (# of newly developed symptoms) are being assessed but what is the third variable (i.e., mediator)? Reading on, I realize that the exposure (vaccination) and outcome (prolonged symptoms) are being examined in the presence of severity. Would suggest revising this section to be clearer as this is not evident without substantial consideration.

In addition, simply adding or removing the variable associated with severity may not equate to a mediation analysis. This sounds to me more like a sensitivity analysis. Would recommend reconsidering methodology

Apologies that this section was not clear and we thank the reviewer for these suggestions. For clarification we added a supplementary figure with a DAG and changed this section. The reviewer is right that this is not a formal mediation analysis. We chose to rephrase it into 'assessing the possibility of a mediating effect' instead of performing an analysis into the (in)direct effects.

Line 185: For the association between vaccination and new post-acute COVID symptoms at 100 days after test result we estimated odds ratios (adjusted for sex, age, livability index and period of infection). The primary aim of vaccination is to prevent severe COVID-19. It is likely that the mechanism by which vaccination affects persistence of post-acute COVID symptoms, might be through the effect of vaccination on severity of acute disease (mediation, see supplementary figure 2). To investigate this, we first used linear regression to assess the association between vaccination and number of newly developed acute symptoms (severity) and secondly we used logistic regression to assess the association between number of newly developed acute symptoms and persistence of new post-acute COVID symptoms. Subsequently, the possibility of a mediating effect of severity of disease in the association between vaccination and persistence of new post-acute COVID symptoms was assessed by adding the number of newly developed acute symptoms to the model and compare the association between vaccination and persistence of new post-acute COVID symptoms with and without this adjustment. A difference between these associations indicates a possible mediating effect.

Line 275: Including the number of newly developed symptoms during the acute phase (as a proxy for severity of acute infection) the association between vaccination and post-acute COVID symptoms at 100 days, changed from 0.6 to 0.7, which implies that severity of disease is a possible mediator in the effect of vaccination on post-acute COVID symptoms.

Results:

- The description of the eligible cohort is exceptional. Very elegant. It is particularly helpful to understand the characteristics of those who were excluded.

Thank you for this compliment.

- The subheadings are very clear - would recommend simplifying the research questions in the introduction to objectives that mirror these subheadings.

Thank you for this compliment. We tried to make this even more clear.

- Table 1: suggest replacing "moment" with "time"

We changed this.

Discussion:

- Well considered. Limitations are appropriately oriented in strengths.

Thank you for this compliment.

1. Von Elm, E., et al., The Strengthening the Reporting of Observational Studies in Epidemiology (STROBE) statement: guidelines for reporting observational studies. The lancet, 2007. 370(9596): p. 1453-1457.

2. Rosendaal, F., The p-value: A clinician's disease? European Journal of Internal Medicine, 2016. 35: p. 20-23.

3. Harrington, D., et al., New guidelines for statistical reporting in the journal. 2019, Mass Medical Soc. p. 285-286.

VERSION 2 - REVIEW

**REVIEWER NAME**	Sharath, Sherene E.
**REVIEWER AFFILIATION**	SUNY Downstate Health Sciences University, Department of Surgery
**REVIEWER CONFLICT OF INTEREST**	No competing interests.
**DATE REVIEW RETURNED**	20-Aug-2024

**GENERAL COMMENTS**	Thank you for your time and effort in considering my suggestions. Congratulations again on your work.
